# Supplementary material for: A single power stroke by ATP binding drives substrate translocation in a heterodimeric ABC transporter
Source: eLife. 2020 Apr 21;9:e55943. doi: 10.7554/eLife.55943 (PMC7205462; doi:10.7554/eLife.55943)
Supplement: Figure 1—source data 1. [file elife-55943-fig1-data1.docx]

| Figure 1 | b |  |  |  |  |
| --- | --- | --- | --- | --- | --- |
|  |  | Sample |  | mean | sd |
|  |  |  |  | cpm*10^3 | cpm*10^3 |
|  |  |  |  |  |  |
|  |  | No ATP |  | 35.53 | 2.35 |
|  |  | ATP |  | 1.47 | 3.35 |

| Figure 1 | c |  |  |  |  |
| --- | --- | --- | --- | --- | --- |
|  |  | Sample |  | mean | sd |
|  |  |  |  | Fluorescence | Fluorescence |
|  |  |  |  | anisotropy | anisotropy |
|  |  |  |  |  |  |
|  |  | No TmrA(EQ)B | | 0.0231 | 0.0006 |
|  |  | No ATP |  | 0.0459 | 0.0010 |
|  |  | TmrA(EQ)B+ATP | | 0.0240 | 0.0001 |

| Figure 1 | d |  |  |  |  |  |
| --- | --- | --- | --- | --- | --- | --- |
|  |  | Sample |  |  |  |  |
|  |  |  |  | log10 ATP | mean | sd |
|  |  |  |  | M | % | % |
|  |  |  |  |  |  |  |
|  |  |  |  | -9 | 96.50 | 1.92 |
|  |  |  |  | -6 | 100.00 | 1.67 |
|  |  |  |  | -5 | 81.57 | 1.87 |
|  |  |  |  | -4.522879 | 45.85 | 1.65 |
|  |  |  |  | -4 | 23.92 | 1.80 |
|  |  |  |  | -3.522879 | 10.81 | 1.67 |
|  |  |  |  | -3 | 6.31 | 1.70 |
|  |  |  |  | -2.30103 | 6.49 | 1.71 |
|  |  |  |  |  |  |  |
|  |  |  |  |  |  |  |
|  |  | Sample |  | log10 ADP | mean | sd |
|  |  |  |  | M | % | % |
|  |  |  |  |  |  |  |
|  |  |  |  | -6 | 96.50 | 1.92 |
|  |  |  |  | -5 | 100.00 | 1.62 |
|  |  |  |  | -3 | 84.86 | 1.63 |

| Figure 1 | e |  |  |  |  |  |
| --- | --- | --- | --- | --- | --- | --- |
|  |  | Sample |  | ATP | mean | sd |
|  |  |  |  | mM | cpm*10^3 | cpm*10^3 |
|  |  |  |  |  |  |  |
|  |  |  |  | 0.003 | 133.06 | 1.88 |
|  |  |  |  | 0.01 | 311.54 | 6.56 |
|  |  |  |  | 0.03 | 556.61 | 16.50 |
|  |  |  |  | 0.1 | 1006.60 | 23.79 |
|  |  |  |  | 0.3 | 1695.40 | 193.37 |
|  |  |  |  |  |  |  |
|  |  |  |  |  |  |  |
|  |  | Sample |  | ADP | mean | sd |
|  |  |  |  | mM | cpm*10^3 | cpm*10^3 |
|  |  |  |  |  |  |  |
|  |  |  |  | 0.003 | 96.85 | 2.74 |
|  |  |  |  | 0.01 | 176.91 | 3.41 |
|  |  |  |  | 0.03 | 389.99 | 5.75 |
|  |  |  |  | 0.1 | 696.20 | 23.36 |
|  |  |  |  | 0.3 | 1505.40 | 48.91 |

| Figure 1 - figure supplement 1 | | | b |  |  |  |
| --- | --- | --- | --- | --- | --- | --- |
|  |  |  | Peptide |  | mean | sd |
|  |  |  | µM |  | cpm*10^3 | cpm*10^3 |
|  |  |  |  |  |  |  |
|  |  |  | 0.01 |  | 0.040 | 0.033 |
|  |  |  | 0.1 |  | 0.170 | 0.094 |
|  |  |  | 0.3 |  | 0.627 | 0.363 |
|  |  |  | 1 |  | 3.293 | 0.381 |
|  |  |  | 3 |  | 8.853 | 0.610 |
|  |  |  | 10 |  | 28.000 | 6.632 |
|  |  |  | 30 |  | 63.867 | 16.760 |
|  |  |  | 100 |  | 126.333 | 26.173 |

| Figure 1 - figure supplement 1 | | | c |  |  |  |
| --- | --- | --- | --- | --- | --- | --- |
|  |  |  | Sample |  | mean | sd |
|  |  |  |  |  | cpm | cpm |
|  |  |  |  |  |  |  |
|  |  |  | WT | peptide binding | 3434.00 | 46.77 |
|  |  |  | WT | background | 1672.67 | 42.55 |
|  |  |  | EQ | peptide binding | 3348.33 | 16.04 |
|  |  |  | EQ | background | 1933.33 | 56.00 |

| Figure 1 - figure supplement 1 | | | d |  |  |  |
| --- | --- | --- | --- | --- | --- | --- |
|  |  |  | Sample |  | mean | sd |
|  |  |  |  |  | cpm | cpm |
|  |  |  |  |  |  |  |
|  |  |  | WT | ATP binding | 6379.00 | 126.82 |
|  |  |  | WT | background | 1154.33 | 41.96 |
|  |  |  | EQ | ATP binding | 6423.00 | 82.16 |
|  |  |  | EQ | background | 1274.00 | 20.42 |

| Figure 1 - figure supplement 2 | | | a |  |  |  |
| --- | --- | --- | --- | --- | --- | --- |
|  |  |  | Sample |  | mean | sd |
|  |  |  |  |  | cpm*10^3 | cpm*10^3 |
|  |  |  |  |  |  |  |
|  |  |  | No ATPgS |  | 20.07 | 0.13 |
|  |  |  | ATPgS |  | 2.47 | 1.28 |

| Figure 1 - figure supplement 2 | | | b |  |  |  |  |
| --- | --- | --- | --- | --- | --- | --- | --- |
|  |  |  | Sample |  |  |  |  |
|  |  |  |  |  | log10 ATP | mean | sd |
|  |  |  |  |  | M | % | % |
|  |  |  |  |  |  |  |  |
|  |  |  | ATP |  | -9.00 | 100.00 | 14.09 |
|  |  |  | ATP |  | -5.00 | 87.56 | 13.43 |
|  |  |  | ATP |  | -4.52 | 59.18 | 10.24 |
|  |  |  | ATP |  | -3.52 | 37.61 | 7.09 |
|  |  |  | ATP |  | -3.00 | 21.48 | 5.83 |
|  |  |  |  |  |  |  |  |
|  |  |  |  |  |  |  |  |
|  |  |  | Sample |  |  |  |  |
|  |  |  |  |  | log10 ADP | mean | sd |
|  |  |  |  |  | M | % | % |
|  |  |  |  |  |  |  |  |
|  |  |  | ADP |  | -6.00 | 97.87 | 8.05 |
|  |  |  | ADP |  | -4.52 | 100.00 | 4.96 |
|  |  |  | ADP |  | -3.00 | 92.13 | 8.44 |
