# Supplementary material for: A single power stroke by ATP binding drives substrate translocation in a heterodimeric ABC transporter
Source: eLife. 2020 Apr 21;9:e55943. doi: 10.7554/eLife.55943 (PMC7205462; doi:10.7554/eLife.55943)
Supplement: Figure 2—source data 1. [file elife-55943-fig2-data1.docx]

| Figure 2 | c |  |  |  |  |
| --- | --- | --- | --- | --- | --- |
|  |  | Sample |  | mean | sd |
|  |  |  |  | 1/min | 1/min |
|  |  |  |  |  |  |
|  |  | TmrA(EQ)B+ATP | | 0.0338 | 0.0021 |
|  |  | TmrA(EQ)B+EDTA | | 0.0000 | 0.0019 |
|  |  | ATP alone |  | 0.0009 | 0.0028 |

| Figure 2 | d |  |  |  |  |
| --- | --- | --- | --- | --- | --- |
|  |  | Time |  | mean | sd |
|  |  | min |  | cpm*10^3 | cpm*10^3 |
|  |  |  |  |  |  |
|  |  | 0.00 |  | 9.87 | 0.36 |
|  |  | 7.00 |  | 8.48 | 0.34 |
|  |  | 13.98 |  | 6.94 | 0.25 |
|  |  | 19.98 |  | 5.82 | 0.10 |
|  |  | 27.00 |  | 5.19 | 0.17 |
|  |  | 34.00 |  | 4.55 | 0.36 |
|  |  | 41.00 |  | 4.19 | 0.20 |
|  |  | 47.00 |  | 3.69 | 0.26 |
|  |  | 54.00 |  | 3.69 | 0.25 |
|  |  | 61.00 |  | 3.50 | 0.15 |
|  |  | 67.98 |  | 3.09 | 0.35 |
|  |  | 73.98 |  | 3.17 | 0.12 |
|  |  | 81.00 |  | 2.94 | 0.21 |
|  |  | 88.02 |  | 2.88 | 0.13 |
|  |  | 94.02 |  | 2.58 | 0.12 |
|  |  | 101.00 |  | 2.56 | 0.11 |
|  |  | 108.00 |  | 2.64 | 0.16 |
|  |  | 115.00 |  | 2.44 | 0.25 |
|  |  | 120.96 |  | 2.22 | 0.18 |
|  |  | 127.98 |  | 2.24 | 0.13 |
|  |  | 135.00 |  | 2.11 | 0.19 |
|  |  | 142.02 |  | 2.05 | 0.10 |
|  |  | 148.00 |  | 2.01 | 0.11 |
|  |  | 155.00 |  | 1.84 | 0.18 |
|  |  | 162.00 |  | 1.87 | 0.14 |
|  |  | 168.00 |  | 1.81 | 0.23 |
|  |  | 175.00 |  | 1.69 | 0.23 |
|  |  | 181.98 |  | 1.81 | 0.23 |

| Figure 2 | e |  |  |  |  |  |
| --- | --- | --- | --- | --- | --- | --- |
|  |  |  |  |  |  |  |
|  |  | Sample | Time |  | mean | sd |
|  |  |  | min |  | cpm*10^3 | cpm*10^3 |
|  |  |  |  |  |  |  |
|  |  | No ATP | 0.00 |  | 35.53 | 2.35 |
|  |  | No ATP | 8.00 |  | 40.36 | 2.46 |
|  |  | No ATP | 16.00 |  | 43.56 | 2.81 |
|  |  | No ATP | 25.00 |  | 44.72 | 2.46 |
|  |  | No ATP | 31.00 |  | 47.00 | 2.58 |
|  |  | No ATP | 38.00 |  | 46.81 | 2.26 |
|  |  | No ATP | 45.00 |  | 47.97 | 3.50 |
|  |  | No ATP | 52.02 |  | 49.36 | 3.73 |
|  |  | No ATP | 58.00 |  | 49.24 | 2.78 |
|  |  | No ATP | 65.00 |  | 50.61 | 2.49 |
|  |  | No ATP | 72.00 |  | 50.28 | 2.63 |
|  |  | No ATP | 79.00 |  | 51.85 | 2.60 |
|  |  | No ATP | 85.00 |  | 50.29 | 3.42 |
|  |  | No ATP | 103.98 |  | 54.07 | 3.68 |
|  |  | No ATP | 111.00 |  | 53.61 | 2.61 |
|  |  | No ATP | 117.00 |  | 55.47 | 2.26 |
|  |  | No ATP | 124.02 |  | 53.35 | 3.89 |
|  |  | No ATP | 130.98 |  | 55.16 | 2.29 |
|  |  | No ATP | 138.00 |  | 55.08 | 2.46 |
|  |  | No ATP | 144.00 |  | 56.32 | 2.34 |
|  |  | No ATP | 151.00 |  | 56.89 | 3.04 |
|  |  | No ATP | 158.00 |  | 56.21 | 2.72 |
|  |  | No ATP | 165.00 |  | 55.56 | 2.51 |
|  |  | No ATP | 172.02 |  | 56.60 | 2.33 |
|  |  |  |  |  |  |  |
|  |  |  |  |  |  |  |
|  |  | Sample | Time |  | mean | sd |
|  |  |  | min |  | cpm*10^3 | cpm*10^3 |
|  |  |  |  |  |  |  |
|  |  | ATP | 0.00 |  | -1.91 | 2.77 |
|  |  | ATP | 8.00 |  | 12.73 | 4.19 |
|  |  | ATP | 16.00 |  | 20.39 | 2.32 |
|  |  | ATP | 25.00 |  | 27.25 | 1.87 |
|  |  | ATP | 31.00 |  | 31.92 | 3.24 |
|  |  | ATP | 38.00 |  | 35.35 | 3.36 |
|  |  | ATP | 45.00 |  | 36.56 | 2.71 |
|  |  | ATP | 52.02 |  | 39.71 | 2.18 |
|  |  | ATP | 58.00 |  | 41.97 | 3.45 |
|  |  | ATP | 65.00 |  | 42.57 | 2.82 |
|  |  | ATP | 72.00 |  | 45.72 | 5.18 |
|  |  | ATP | 79.00 |  | 45.21 | 2.23 |
|  |  | ATP | 85.00 |  | 45.75 | 3.37 |
|  |  | ATP | 103.98 |  | 49.95 | 3.03 |
|  |  | ATP | 111.00 |  | 49.67 | 3.99 |
|  |  | ATP | 117.00 |  | 48.81 | 4.49 |
|  |  | ATP | 124.02 |  | 50.95 | 3.30 |
|  |  | ATP | 130.98 |  | 50.72 | 4.46 |
|  |  | ATP | 138.00 |  | 50.19 | 4.92 |
|  |  | ATP | 144.00 |  | 50.36 | 3.25 |
|  |  | ATP | 151.00 |  | 50.61 | 3.73 |
|  |  | ATP | 158.00 |  | 51.25 | 2.72 |
|  |  | ATP | 165.00 |  | 51.41 | 2.82 |
|  |  | ATP | 172.02 |  | 51.61 | 3.34 |

| Figure 2 | f |  |  |  |  |  |  |
| --- | --- | --- | --- | --- | --- | --- | --- |
|  |  | Sample |  | Time |  | mean | sd |
|  |  |  |  | min |  | % | % |
|  |  |  |  |  |  |  |  |
|  |  | ATP |  | 0.00 |  | 88.31 | 1.04 |
|  |  | ATP |  | 1.00 |  | 87.11 | 0.53 |
|  |  | ATP |  | 5.00 |  | 81.24 | 1.01 |
|  |  | ATP |  | 10.00 |  | 75.44 | 0.67 |
|  |  | ATP |  | 30.00 |  | 64.14 | 1.21 |
|  |  | ATP |  | 60.00 |  | 63.52 | 0.85 |
|  |  | ATP |  | 120.00 |  | 56.57 | 0.83 |
|  |  |  |  |  |  |  |  |
|  |  |  |  |  |  |  |  |
|  |  | Sample |  | Time |  | mean | sd |
|  |  |  |  | min |  | % | % |
|  |  |  |  |  |  |  |  |
|  |  | gamma phosphate | | 0.00 |  | 11.69 | 1.04 |
|  |  | gamma phosphate | | 1.00 |  | 12.89 | 0.53 |
|  |  | gamma phosphate | | 5.00 |  | 18.76 | 1.01 |
|  |  | gamma phosphate | | 10.00 |  | 24.56 | 0.67 |
|  |  | gamma phosphate | | 30.00 |  | 35.86 | 1.21 |
|  |  | gamma phosphate | | 60.00 |  | 36.48 | 0.85 |
|  |  | gamma phosphate | | 120.00 |  | 43.43 | 0.83 |

| Figure 2 - figure supplement 1 | | | a |  |  |  |  |
| --- | --- | --- | --- | --- | --- | --- | --- |
|  |  |  | Sample | Time |  | mean | sd |
|  |  |  |  | min |  | cpm*10^3 | cpm*10^3 |
|  |  |  |  |  |  |  |  |
|  |  |  | ATP (20 °C) | 0.00 |  | 5.73 | 0.19 |
|  |  |  | ATP (20 °C) | 7.00 |  | 4.59 | 0.09 |
|  |  |  | ATP (20 °C) | 13.00 |  | 3.75 | 0.11 |
|  |  |  | ATP (20 °C) | 19.98 |  | 3.14 | 0.20 |
|  |  |  | ATP (20 °C) | 27.00 |  | 2.75 | 0.12 |
|  |  |  | ATP (20 °C) | 34.00 |  | 2.15 | 0.17 |
|  |  |  | ATP (20 °C) | 40.00 |  | 2.14 | 0.14 |
|  |  |  | ATP (20 °C) | 47.00 |  | 2.12 | 0.16 |
|  |  |  | ATP (20 °C) | 54.00 |  | 1.96 | 0.10 |
|  |  |  | ATP (20 °C) | 61.00 |  | 1.76 | 0.14 |
|  |  |  | ATP (20 °C) | 67.00 |  | 1.60 | 0.16 |
|  |  |  | ATP (20 °C) | 74.00 |  | 1.56 | 0.14 |
|  |  |  | ATP (20 °C) | 81.00 |  | 1.39 | 0.17 |
|  |  |  | ATP (20 °C) | 88.00 |  | 1.34 | 0.08 |
|  |  |  | ATP (20 °C) | 94.00 |  | 1.31 | 0.14 |
|  |  |  | ATP (20 °C) | 101.00 |  | 1.21 | 0.09 |
|  |  |  | ATP (20 °C) | 108.00 |  | 1.23 | 0.24 |
|  |  |  | ATP (20 °C) | 115.00 |  | 1.16 | 0.10 |
|  |  |  | ATP (20 °C) | 121.00 |  | 1.00 | 0.09 |
|  |  |  | ATP (20 °C) | 128.00 |  | 1.05 | 0.12 |
|  |  |  | ATP (20 °C) | 135.00 |  | 0.94 | 0.08 |
|  |  |  | ATP (20 °C) | 141.00 |  | 0.93 | 0.09 |
|  |  |  | ATP (20 °C) | 148.00 |  | 1.01 | 0.16 |
|  |  |  | ATP (20 °C) | 155.00 |  | 0.96 | 0.10 |
|  |  |  | ATP (20 °C) | 162.00 |  | 0.94 | 0.14 |
|  |  |  | ATP (20 °C) | 168.00 |  | 0.74 | 0.14 |
|  |  |  | ATP (20 °C) | 175.00 |  | 0.71 | 0.11 |
|  |  |  | ATP (20 °C) | 181.00 |  | 0.87 | 0.13 |
|  |  |  |  |  |  |  |  |
|  |  |  |  |  |  |  |  |
|  |  |  | Sample | Time |  | mean | sd |
|  |  |  |  | min |  | cpm*10^3 | cpm*10^3 |
|  |  |  |  |  |  |  |  |
|  |  |  | ATP (4 °C) | 0.00 |  | 0.69 | 0.06 |
|  |  |  | ATP (4 °C) | 6.00 |  | 0.72 | 0.09 |
|  |  |  | ATP (4 °C) | 13.00 |  | 0.46 | 0.07 |
|  |  |  | ATP (4 °C) | 20.00 |  | 0.34 | 0.06 |
|  |  |  | ATP (4 °C) | 27.00 |  | 0.33 | 0.09 |
|  |  |  | ATP (4 °C) | 33.00 |  | 0.30 | 0.02 |
|  |  |  | ATP (4 °C) | 40.00 |  | 0.28 | 0.02 |
|  |  |  | ATP (4 °C) | 47.00 |  | 0.29 | 0.06 |
|  |  |  | ATP (4 °C) | 54.00 |  | 0.21 | 0.02 |
|  |  |  | ATP (4 °C) | 60.00 |  | 0.19 | 0.08 |
|  |  |  | ATP (4 °C) | 67.00 |  | 0.25 | 0.10 |
|  |  |  | ATP (4 °C) | 74.00 |  | 0.25 | 0.04 |
|  |  |  | ATP (4 °C) | 80.00 |  | 0.20 | 0.09 |
|  |  |  | ATP (4 °C) | 87.00 |  | 0.22 | 0.10 |
|  |  |  | ATP (4 °C) | 94.00 |  | 0.24 | 0.03 |
|  |  |  | ATP (4 °C) | 101.00 |  | 0.16 | 0.06 |
|  |  |  | ATP (4 °C) | 107.00 |  | 0.26 | 0.03 |
|  |  |  | ATP (4 °C) | 114.00 |  | 0.23 | 0.03 |
|  |  |  | ATP (4 °C) | 121.00 |  | 0.23 | 0.03 |
|  |  |  | ATP (4 °C) | 128.00 |  | 0.19 | 0.09 |
|  |  |  | ATP (4 °C) | 134.00 |  | 0.23 | 0.10 |
|  |  |  | ATP (4 °C) | 141.00 |  | 0.23 | 0.04 |
|  |  |  | ATP (4 °C) | 148.00 |  | 0.20 | 0.07 |
|  |  |  | ATP (4 °C) | 155.00 |  | 0.21 | 0.09 |
|  |  |  | ATP (4 °C) | 161.00 |  | 0.22 | 0.03 |
|  |  |  | ATP (4 °C) | 168.00 |  | 0.21 | 0.05 |
|  |  |  | ATP (4 °C) | 175.00 |  | 0.20 | 0.07 |
|  |  |  | ATP (4 °C) | 182.00 |  | 0.12 | 0.02 |

| Figure 2 - figure supplement 1 | | | b |  |  |  |
| --- | --- | --- | --- | --- | --- | --- |
|  |  |  | Time |  | mean | sd |
|  |  |  | min |  | % | % |
|  |  |  |  |  |  |  |
|  |  |  | 0.00 |  | 100.00 | 6.72 |
|  |  |  | 15.00 |  | 83.45 | 2.60 |
|  |  |  | 40.00 |  | 66.55 | 4.65 |
|  |  |  | 103.00 |  | 46.89 | 4.06 |
|  |  |  | 181.98 |  | 41.24 | 4.45 |

| Figure 2 - figure supplement 1 | | | c |  |  |  |
| --- | --- | --- | --- | --- | --- | --- |
|  |  |  | Time |  | mean | sd |
|  |  |  | min |  | % | % |
|  |  |  |  |  |  |  |
|  |  |  | 0.00 |  | 100.00 | 2.99 |
|  |  |  | 10.00 |  | 73.32 | 4.41 |
|  |  |  | 26.00 |  | 40.62 | 1.34 |
|  |  |  | 44.00 |  | 28.02 | 2.30 |
|  |  |  | 86.00 |  | 13.94 | 1.24 |
|  |  |  | 155.00 |  | 7.55 | 1.09 |
|  |  |  | 214.00 |  | 5.28 | 1.65 |
