# Supplementary material for: A single power stroke by ATP binding drives substrate translocation in a heterodimeric ABC transporter
Source: eLife. 2020 Apr 21;9:e55943. doi: 10.7554/eLife.55943 (PMC7205462; doi:10.7554/eLife.55943)
Supplement: Figure 3—source data 1. [file elife-55943-fig3-data1.docx]

| Figure 3 | b |  |  |  |  |
| --- | --- | --- | --- | --- | --- |
|  |  | Sample |  | mean | sd |
|  |  |  |  | Transported | Transported |
|  |  |  |  | peptide | peptide |
|  |  |  |  | µmol/g | µmol/g |
|  |  |  |  |  |  |
|  |  | ATP |  | 1.116 | 0.015 |
|  |  | ADP |  | 0.394 | 0.006 |

| Figure 3 | c |  |  |  |
| --- | --- | --- | --- | --- |
|  |  | Sample |  |  |
|  |  |  |  | peptide |
|  |  |  |  | pmol |
|  |  |  |  |  |
|  |  | ATP | Fraction 1 | 1.58 |
|  |  | ATP | Fraction 2 | 18.74 |
|  |  | ATP | Fraction 3 | 48.10 |
|  |  | ATP | Fraction 4 | 24.16 |
|  |  | ATP | Fraction 5 | 17.47 |
|  |  | ATP | Fraction 6 | 13.07 |
|  |  | ATP | Fraction 7 | 10.76 |
|  |  | ATP | Fraction 8 | 12.26 |
|  |  | ATP | Fraction 9 | 11.37 |
|  |  |  |  |  |
|  |  | ADP | Fraction 1 | 1.22 |
|  |  | ADP | Fraction 2 | 9.24 |
|  |  | ADP | Fraction 3 | 9.19 |
|  |  | ADP | Fraction 4 | 13.73 |
|  |  | ADP | Fraction 5 | 10.31 |
|  |  | ADP | Fraction 6 | 8.69 |
|  |  | ADP | Fraction 7 | 8.07 |
|  |  | ADP | Fraction 8 | 8.17 |
|  |  | ADP | Fraction 9 | 9.02 |
