# Supplementary material for: A single power stroke by ATP binding drives substrate translocation in a heterodimeric ABC transporter
Source: eLife. 2020 Apr 21;9:e55943. doi: 10.7554/eLife.55943 (PMC7205462; doi:10.7554/eLife.55943)
Supplement: Figure 4—figure supplement 1—source data 1. [file elife-55943-fig4-figsupp1-data1.docx]

| Figure 4 - figure supplement 1 | | | b |  |  |  |
| --- | --- | --- | --- | --- | --- | --- |
|  |  |  | Peptides per | | mean | sd |
|  |  |  | liposome |  | Mean fluorescence | Mean fluorescence |
|  |  |  |  |  | intensity | intensity |
|  |  |  |  |  |  |  |
|  |  |  | 0.00 |  | 0.00 | 0.00 |
|  |  |  | 10.78 |  | 439.10 | 11.32 |
|  |  |  | 42.27 |  | 2559.10 | 341.46 |
|  |  |  | 90.77 |  | 5470.77 | 247.01 |
