# Supplementary material for: A single power stroke by ATP binding drives substrate translocation in a heterodimeric ABC transporter
Source: eLife. 2020 Apr 21;9:e55943. doi: 10.7554/eLife.55943 (PMC7205462; doi:10.7554/eLife.55943)
Supplement: Figure 5—source data 1. [file elife-55943-fig5-data1.docx]

| Figure 5 | c |  |  |  |  |
| --- | --- | --- | --- | --- | --- |
|  |  | Sample | Time | mean | sd |
|  |  |  | min | Peptides per | Peptides per |
|  |  |  |  | liposome | liposome |
|  |  |  |  |  |  |
|  |  | ATP | 2 | 4.53 | 0.22 |
|  |  | ATP | 5 | 14.04 | 0.59 |
|  |  | ATP | 10 | 29.02 | 1.01 |
|  |  | ATP | 20 | 38.55 | 0.71 |
|  |  | ATP | 40 | 55.47 | 0.51 |
|  |  |  |  |  |  |
|  |  |  |  |  |  |
|  |  | Sample | Time | mean | sd |
|  |  |  | min | Peptides per | Peptides per |
|  |  |  |  | liposome | liposome |
|  |  |  |  |  |  |
|  |  | ADP | 5 | 0.93 | 0.21 |
|  |  | ADP | 40 | 7.20 | 0.59 |

| Figure 5 | d |  |  |  |  |  |
| --- | --- | --- | --- | --- | --- | --- |
|  |  | Sample | Switch | Return | mean | sd |
|  |  |  |  |  | Peptides per | Peptides per |
|  |  |  |  |  | liposome | liposome |
|  |  |  |  |  |  |  |
|  |  | ATP | 1 | 0 | 42.09 | 1.04 |
|  |  | ATP | 1 | 1 | 38.39 | 0.53 |
|  |  | ATP | 2 | 1 | 89.60 | 1.39 |
|  |  | ATP | 2 | 2 | 95.41 | 0.65 |
|  |  | ADP | 2 | 2 | 11.95 | 0.21 |

| Figure 5 - figure supplement 1 | | | c |  |  |  |
| --- | --- | --- | --- | --- | --- | --- |
|  |  |  | Sample |  | mean | sd |
|  |  |  |  |  | Mean fluorescence | Mean fluorescence |
|  |  |  |  |  | intensity | intensity |
|  |  |  |  |  | C4AF647 | C4AF647 |
|  |  |  |  |  |  |  |
|  |  |  | ADP |  | 1696.667 | 34.53018 |
|  |  |  | ATP |  | 2083 | 26.21069 |
|  |  |  |  |  |  |  |
|  |  |  |  |  |  |  |
|  |  |  | Sample |  | mean | sd |
|  |  |  |  |  | Mean fluorescence | Mean fluorescence |
|  |  |  |  |  | intensity | intensity |
|  |  |  |  |  | C4F | C4F |
|  |  |  |  |  |  |  |
|  |  |  | ADP |  | 131.6667 | 9.865766 |
|  |  |  | ATP |  | 198.6667 | 6.110101 |
